# Supplementary material for: In Silico Insights into Protein–Protein Interaction Disruptive Mutations in the PCSK9-LDLR Complex
Source: Int J Mol Sci. 2020 Feb 25;21(5):1550. doi: 10.3390/ijms21051550 (PMC7084799; doi:10.3390/ijms21051550)
Supplement: Supplementary file 1 [file ijms-21-01550-s001.pdf]

Supplementary Information

Title: ***In Silico* Insights into Protein-Protein Interaction Disruptive Mutations in PCSK9 and LDLR complex**

Corresponding to Feixiong Cheng, Ph.D.

Lerner Research Institute, Cleveland Clinic

Email: chengf@ccf.org

Fax: 216-6361609; Tel: 216-4447654

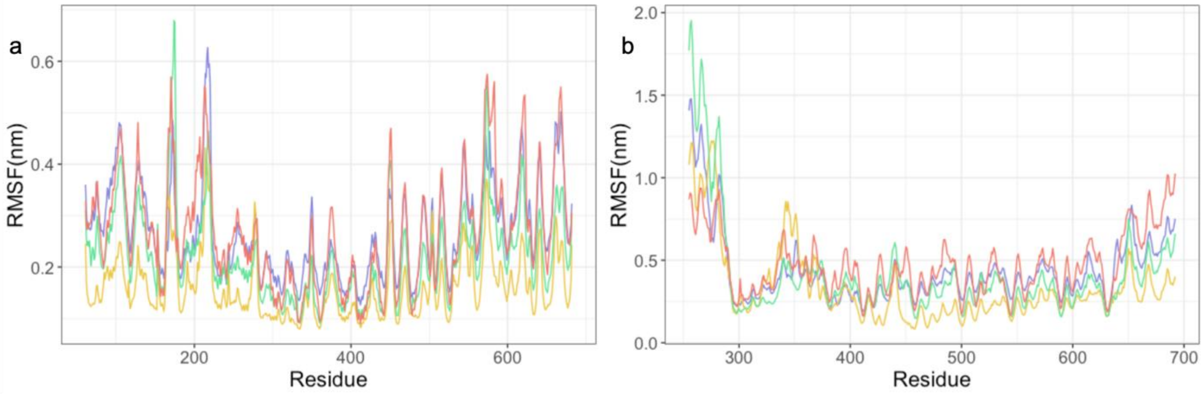

Supplementary Figure S1: RMSF plots of (a) PCSK9 and (b) LDLR. Wild-type is colored in purple, Ser127Arg in green, Asp374Tyr in yellow, and Arg496Trp in red.

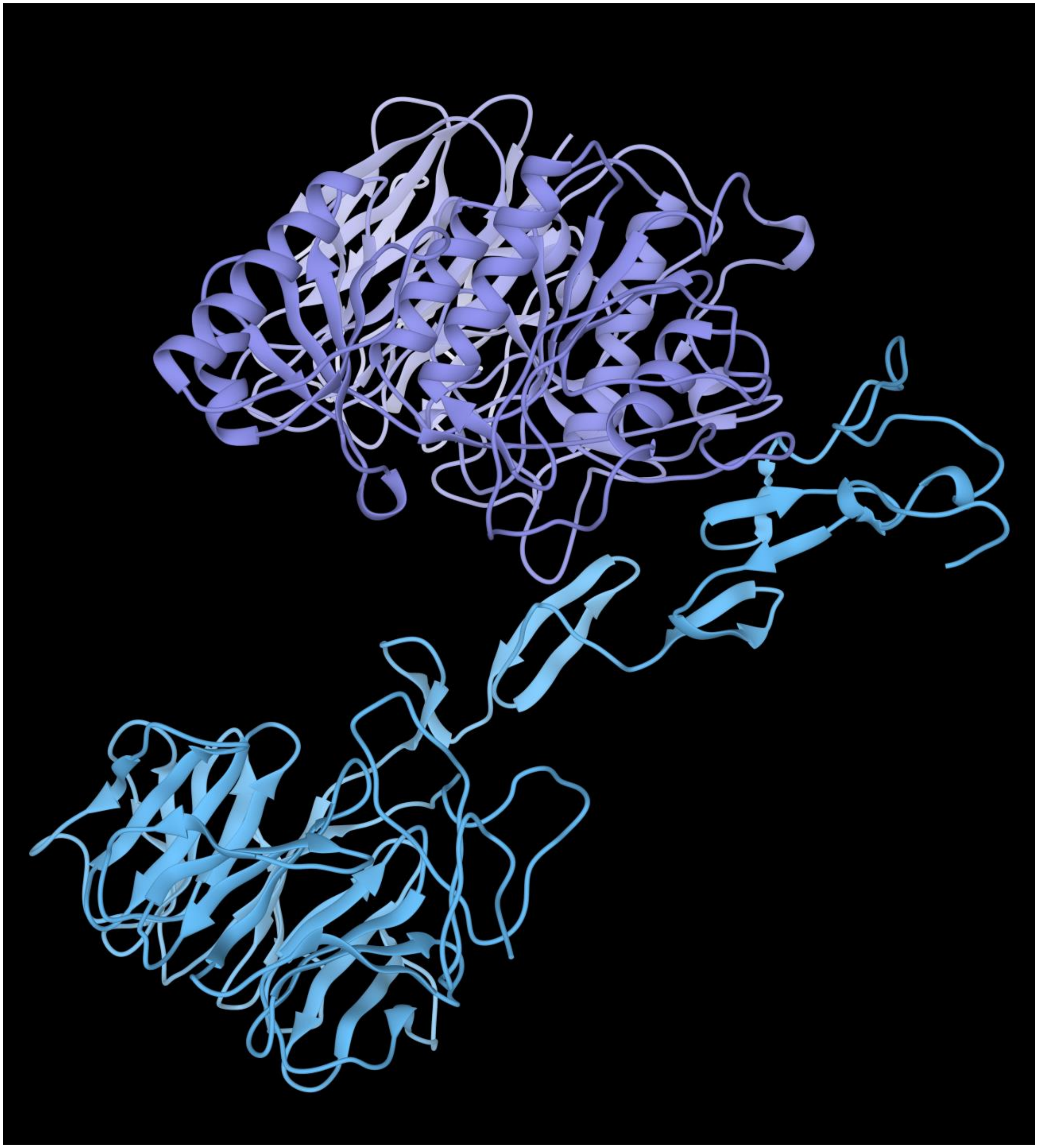

Supplemental Figure S2: A replicate simulation of the Arg496Trp mutant in which the PCSK9/LDLR interaction does not involve the prodomain/β-propeller interface, but instead maintains an extended conformation.

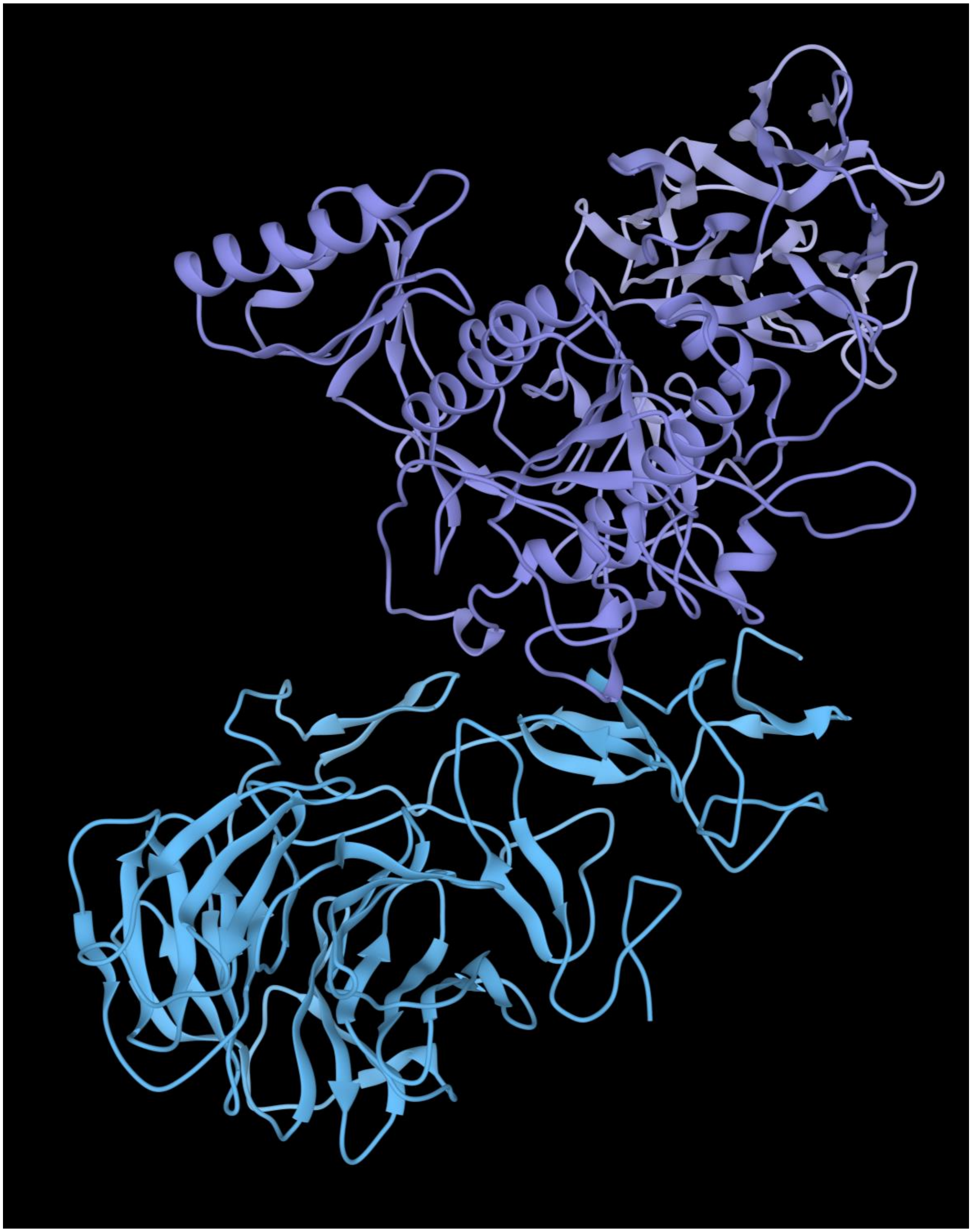

Supplemental Figure S3: Final conformation of a replicate simulation of the Asp374Tyr mutant, showing the C terminus of the extracellular portion of the LDL receptor interacting with the EGF(A) domain.

RESIDUE #	Wild Type	Asp374Tyr	Ser127Arg	Arg496Trp
**70**	4	3	0	0
**71**	6	4	0	0
**73**	1	2	1	0
**74**	1	0	1	1
**75**	3	2	4	2
**76**	0	0	2	2
**78**	0	0	1	0
**108**	0	0	3	3
**110**	0	0	2	3
**112**	0	0	1	0
**125**	0	0	2	2
**126**	0	0	1	1
**127**	0	0	4	4
**145**	0	0	1	0

Supplementary Table S1: Data used to generate the heatmap for the prodomain of PCSK9/LDLR interface region. No residues from the PDB met the criteria for contact used.

RESIDUE #	PDB	Wild Type	Asp374Tyr	Ser127Arg	Arg496Trp
**369**	2	3	2	3	2
**372**	1	0	0	0	0
**374**	1	0	3	0	1
**375**	1	1	1	1	1
**377**	2	2	2	2	2
**378**	4	4	3	4	4
**379**	5	5	6	4	5
**380**	1	3	2	3	2
**381**	0	1	2	2	2

Supplementary Table S2: Data used to generate the heatmap for the crystallized interaction between PCSK9 and LDLR.
